# Reframing Professionalism Through Executive Function: A Multi-Institutional Analysis of Professionalism Lapses in Medical Education

**DOI:** 10.1007/s40670-026-02912-3

**Published:** 2026-10-14

**Authors:** Asia T. Bright, Abbey M. Bachmann, Noor Rejjal, Peggy Hsieh, Vineeth John

**Affiliations:** 1 Office of Professionalism, McGovern Medical School at the University of Texas Health Science Center at Houston, 6431 Fannin Street Suite G.150, Houston, TX 77030 USA; 2 Office of Educational Programs,, McGovern Medical School at the University of Texas Health Science Center at Houston, Houston, TX USA

**Keywords:** Professionalism, Executive functions, Developmental psychology, Competency based, Medical education

## Abstract

**Background:**

Professionalism is a core competency in medical education, yet challenges persist in translating professionalism expectations into observable, assessable, and teachable behaviors. Executive function (EF), a set of cognitive processes that support planning, self-regulation, emotional control, and adaptive behavior, may offer a complementary framework for understanding how professional behaviors may be enacted in practice.

**Methods:**

We conducted a multi-institutional mixed-methods exploratory study examining professionalism concerns through an EF lens. Medical educators were surveyed regarding professionalism lapses observed among learners in undergraduate and graduate medical education. Responses from 80 participants representing 64 institutions were coded into predefined EF domains using a structured coding framework. Descriptive analyses examined EF frequencies, regional distributions, and co-occurring EF domains. Open-ended responses underwent reflexive thematic analysis to explore broader perspectives regarding professionalism.

**Results:**

Participants reported 425 professionalism concerns, yielding 646 EF coding occurrences. The most frequently identified EF domains were metacognition (18.0%), planning/prioritizing (17.0%), response inhibition (14.4%), and emotional control (13.5%). Common EF pairings included emotional control with response inhibition and metacognition with planning/prioritizing. Reflexive thematic analysis of 30 open-ended responses identified five themes: Professionalism as a Socio-Cultural Construct, Longstanding Professionalism Challenges Within Medical Education, Professionalism Behaviors Consistent with Executive Function Processes, Modern Professionalism Challenges, and the COVID-19 Pandemic as an Inflection Point.

**Conclusions:**

Professionalism concerns clustered around identifiable EF domains. An EF-informed framework may help operationalize professionalism by linking expectations to observable behaviors and may support more specific feedback, coaching, and assessment while preserving professionalism as a complex socio-cultural construct.

**Supplementary Information:**

The online version contains supplementary material available at https://doi.org/10.1007/s40670-026-02912-3.

## Introduction

Professionalism is a foundational tenet of medicine and central to the professional identity formation of future physicians [1, 2]. Historically, professionalism was conceptualized as a stable set of virtues including integrity, altruism, accountability, and moral character. Expectations were often implicit, shaped by institutional culture and the hidden curriculum, and reinforced through observation and socialization rather than explicit instruction [3]. Over time, professionalism frameworks evolved toward competency-based models emphasizing communication, teamwork, adaptability, cultural humility, social responsibility, and digital professionalism, acknowledging that professional identity and behavior extend beyond face-to-face interactions to include electronic communication, social media engagement, virtual learning environments, and responsible stewardship of digital information [2, 4].

Despite this evolution, professionalism remains one of the most difficult competencies to operationalize and assess consistently. Professionalism now includes a broad and evolving range of expectations that can differ across institutions and contexts, without a single universally agreed-upon framework [2]. Although numerous professionalism frameworks have been proposed, including those developed by the Accreditation Council for Graduate Medical Education (ACGME) and the Association of American Medical Colleges (AAMC), none provides a universally accepted operational approach for translating professionalism expectations into observable behaviors suitable for consistent assessment across contexts. Instead, professionalism remains inherently context dependent, requiring educators to interpret behaviors within institutional, clinical, and cultural environments [1, 2, 5]. As a result, educators often struggle to define, observe, and assess professionalism consistently [5]. This ambiguity contributes to subjectivity and bias in evaluation [6]. Faculty frequently rely on vague or indirect language, leading to inconsistent documentation and limited actionable feedback for learners [5]. Evidence also demonstrates disparities in professionalism assessments, with racially minoritized trainees and women more likely to receive lower ratings or have behaviors interpreted through a lens of personal character rather than observable actions [6]. Consequently, professionalism’s potential as a developmental construct for training excellence is diminished to a mechanism of policing behavior, reinforcing inequities and fostering learner disengagement. This lack of operational specificity provides the rationale for exploring complementary frameworks that may improve behavioral interpretation.

At the same time, developmental psychology and neuroscience suggest that most medical students and early residents are navigating the developmental stage of emerging adulthood, typically spanning ages 18 to 29 [7]. The typical age range for students starting medical school is 22–24 years old and the start of residency is 26–29 years old. This period is characterized by ongoing identity formation, emotional maturation, increasing autonomy, and continued development of executive function (EF) processes within the prefrontal cortex [8]. EF are higher-order cognitive processes that support planning, self-regulation, emotional control, cognitive flexibility, organization, and adaptive behavior [8]. Rather than functioning independently, these processes operate in an integrated and context-dependent manner, shaping how individuals learn, make decisions, respond to feedback, and engage with others. Medical education places extraordinary executive demands on learners. Students and residents must simultaneously prioritize competing clinical tasks, manage uncertainty, coordinate patient care across teams, respond constructively to feedback, regulate emotional responses under stress, and adapt to rapidly changing clinical situations. Successful performance therefore depends not only on medical knowledge but also on the executive processes that support self-regulation and adaptive behavior. Importantly, neuroscientific research demonstrates that EF development continues well into early adulthood (ages 18–29). Although some residents may extend beyond this age range, executive function development remains heterogeneous across individuals, and performance continues to be influenced by stress, workload, and environmental demands throughout early professional training [8, 9]. This developmental variability does not imply that educators should attempt to assess executive function directly. Instead, professionalism assessment should remain focused on observable behaviors. The EF framework is intended to help educators interpret those behaviors and identify potential coaching targets without assuming that executive function deficits are present or are the sole explanation for the observed professionalism concern. Although many learners effectively meet professional expectations, professionalism lapses may become more apparent when situational demands exceed an individual’s current developmental capacity, particularly in high-pressure or unfamiliar settings [8, 9].

Executive function has become increasingly recognized as a foundational construct for understanding performance in complex, dynamic environments [8, 10]. Rather than reflecting isolated cognitive abilities, executive functions coordinate goal-directed behavior by enabling individuals to plan, monitor performance, regulate emotions, inhibit impulsive responses, shift strategies when circumstances change, and learn from feedback. These processes become particularly important in situations characterized by competing demands, uncertainty, and rapidly changing priorities which are all defining features of clinical learning environments.

Collectively, these observations suggest that, many behaviors identified by medical educators as professionalism concerns may be interpreted through an EF lens as they involve cognitive processes related to planning, self-regulation, emotional control, and metacognition. Many behaviors traditionally evaluated as professionalism include punctuality, communication, accountability, teamwork, adaptability, responsiveness to feedback, and follow-through require coordinated executive function processes. For example, arriving prepared for clinical responsibilities requires planning and time management; accepting corrective feedback requires emotional regulation and cognitive flexibility; respectful communication requires inhibitory control and self-monitoring; and maintaining responsibility for patient care requires sustained goal-directed persistence. These observations suggest that executive function may influence how professional expectations are enacted, even though professionalism itself remains a broader sociocultural construct [1–2]. This study does not propose that executive function deficits directly cause professionalism lapses. We propose introducing an executive function (EF) framework (a structured set of cognitive and behavioral domains that support goal-directed behavior) as a complementary lens for interpreting and operationalizing professionalism concerns. This approach reframes professionalism through EF, drawing on developmental and cognitive psychology to create a more precise, equitable, and developmentally aligned framework. Tardiness, incomplete documentation, difficulty incorporating feedback, interpersonal conflict, or lapses in digital professionalism may arise from limitations in planning, inhibitory control, emotional regulation, or metacognitive awareness. Although EF supports many domains beyond professionalism, it may provide a useful framework for operationalizing how professional behaviors may be enacted in complex clinical environments.

Importantly, this framework does not seek to determine whether a behavior is objectively professional or unprofessional. Rather, it begins with behaviors that educators have already identified as professionalism concerns within their institutional and educational contexts. Consistent with the professionalism literature, we recognize that professionalism is a dynamic socio-cultural construct for which no single universally accepted operational framework exists [1–2]. Instead, professional expectations emerge from competency frameworks, institutional policies, accreditation standards, and the norms of clinical practice. The EF framework therefore operates downstream of professionalism judgments. It does not determine whether a behavior is professional; rather, it provides one structured approach for interpreting behaviors that have already been identified as professionalism concerns by educators within their institutional context. The contribution of the executive function framework is therefore not to redefine professionalism, but to provide a structured method for interpreting and discussing observable behaviors once they have been identified as professionalism concerns.

From an educational perspective, executive function offers several advantages as an interpretive framework. Executive function domains are observable, teachable, and potentially modifiable through coaching and structured feedback. Unlike broad professionalism labels, EF provides educators with language that identifies the underlying cognitive and behavioral processes contributing to observed behaviors. This distinction may support more precise feedback while preserving professionalism as the educational outcome rather than redefining professionalism itself. Executive function is not proposed because professionalism is fundamentally cognitive; rather, it is proposed because many observable professionalism behaviors require executive processes to be successfully enacted in practice.

### Current Study

**Fig. 1:**
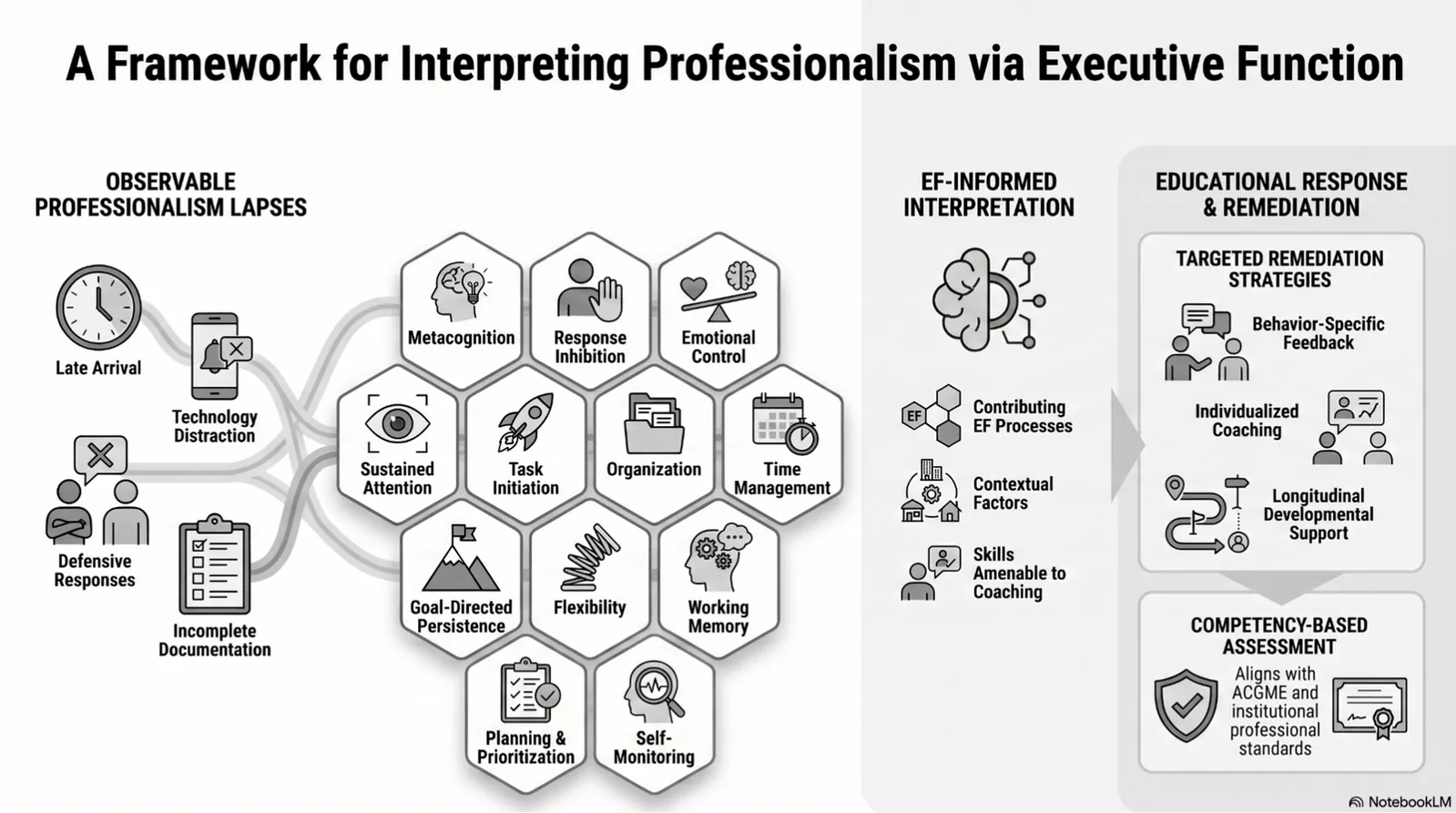
Executive function (EF) as a complementary framework for interpreting professionalism concerns. Professionalism expectations remain defined by competency frameworks, institutional standards, and sociocultural norms. Observable professionalism concerns are interpreted through executive function domains to support more specific feedback, coaching, and remediation. Executive function is proposed as an interpretive framework that informs developmental support rather than as a diagnostic tool or causal explanation for professionalism lapses

The purpose of this study was to examine whether professionalism concerns identified by medical educators could be meaningfully interpreted through an executive function (EF) framework. Our objective was not to develop a new professionalism assessment framework or redefine professionalism itself, but rather to explore whether EF provides a complementary interpretive framework that helps translate observed professionalism concerns into more specific, observable, and developmentally actionable behaviors. Specifically, we sought to identify the most frequently associated EF domains, examine co-occurring EF patterns, and explore variation across geographic regions. By combining conceptual and empirical approaches, this work aims to provide a practical framework for translating broad professionalism expectations into observable, assessable, and teachable behaviors. See Fig. 1.

## Materials

A survey instrument was developed to explore medical educators’ and academic administrators’ perceptions and observations of professionalism concerns among learners in undergraduate and graduate medical education (see Appendix A). Participants were asked to list up to 10 professionalism behaviors they had observed among learners and to indicate whether each behavior occurred in undergraduate medical education (UME), graduate medical education (GME), or both. The upper limit was selected to encourage comprehensive responses while minimizing respondent burden. To capture participants’ natural conceptualizations of professionalism, no predefined professionalism framework or list of professionalism behaviors was provided. Respondents were encouraged to report behaviors they personally considered professionalism concerns based on their educational experiences. Respondents were informed that providing fewer than 10 examples was acceptable and were encouraged to provide as much descriptive detail as desired. An example prompt included: “Tone of voice in email is rude – Both.” (See Appendix A).

The survey also included an optional open-ended question inviting participants to share additional observations or insights that might contribute to understanding professional and unprofessional behaviors in medical education. Demographic questions pertaining to educational administrator characteristics were included following the primary survey items. (See Appendix A)

The survey therefore generated two complementary datasets: (1) structured professionalism behavior responses that were analyzed quantitatively using the executive function coding framework and (2) optional narrative responses that underwent qualitative thematic analysis.

The survey was developed and administered electronically using the QuestionPro virtual survey platform.

## Methods

### Recruitment

Participants were recruited through a medical education DR-ED listserv targeting medical educators and academic administrators involved in undergraduate and graduate medical education. Eligible participants were faculty and administrators with a direct educational role in an accredited UME and/or GME program who had engaged with learners since 2023. The survey invitation included information regarding the purpose of the study and instructions for participation. Participation was voluntary and anonymous, and respondents self-selected into the study.

The anonymous survey remained open for responses for 1 month during September of 2025. Participation was voluntary, and respondents could complete as much or as little of the survey as they wished. No incentives were provided for participation. Responses were collected electronically through the QuestionPro platform. Ethical approval was granted for this study by the Institutional Review Board at the University of Texas Health Science Center at Houston (HSC-MS- 25–0528).

Regional representation was categorized according to the Association of American Medical Colleges (AAMC) Group on Student Affairs (GSA) regional classification system [11]. Institutions located in Illinois, Indiana, Iowa, Kansas, Michigan, Minnesota, Missouri, Nebraska, North Dakota, Ohio, South Dakota, Wisconsin, Manitoba, and Ontario were classified as Central Region schools. Institutions in Connecticut, the District of Columbia, Maine, Massachusetts, New Hampshire, New Jersey, Maryland, New York, Pennsylvania, Rhode Island, Vermont, Newfoundland, Nova Scotia, and Quebec were classified as Northeastern Region schools. Institutions in Alabama, Arkansas, Florida, Georgia, Kentucky, Louisiana, Mississippi, North Carolina, Oklahoma, South Carolina, Tennessee, Texas, Virginia, West Virginia, and Puerto Rico were classified as Southern Region schools. Institutions in Arizona, California, Colorado, Hawaii, Nevada, New Mexico, Oregon, Utah, Washington, Alberta, British Columbia, and Saskatchewan were classified as Western Region schools.

### Data Analysis

This study employed a descriptive, exploratory mixed-methods design to examine professionalism concerns using a predefined executive function (EF) framework. The framework consisted of 11 executive function domains (Table 1), including metacognition, planning/prioritizing, response inhibition, emotional control, time management, goal-directed persistence, flexibility, task initiation, sustained attention, organization, and working memory. These domains served as the conceptual framework through which professionalism concerns reported by educators were coded and interpreted.

**Table 1:** Executive Function Domains and Definitions Note. Definitions adapted from Dawson and Guare’s model of executive skills and supported by contemporary executive function literature. Executive functions are interrelated processes that operate together to support goal-directed behavior, self-regulation, learning, and adaptive performance

Executive Function	Definition
Metacognition	The ability to monitor, evaluate, and adjust one’s own thinking, behavior, and performance. Involves self-awareness, self-reflection, and insight into the impact of one’s actions on others.
Planning/Prioritizing	The ability to identify goals, anticipate future demands, organize tasks, and determine the most effective order for completing activities.
Response Inhibition	The ability to resist impulses, pause before acting, and suppress inappropriate verbal, emotional, or behavioral responses.
Emotional Control	The ability to regulate emotional reactions in order to respond appropriately to situational demands.
Time Management	The ability to estimate, allocate, and monitor time effectively to complete responsibilities and meet deadlines.
Goal-Directed Persistence	The capacity to maintain effort and attention toward long-term goals despite obstacles, frustration, or competing demands.
Flexibility (Cognitive Flexibility)	The ability to adapt to changing circumstances, shift perspectives, consider alternative viewpoints, and modify behavior when needed.
Task Initiation	The ability to begin tasks independently and in a timely manner without excessive procrastination or prompting.
Sustained Attention	The ability to maintain focus and concentration over time despite distractions or competing stimuli.
Organization	The ability to create and maintain systems for managing information, materials, and responsibilities.
Working Memory	The ability to hold, manipulate, and use information in mind while completing cognitive tasks.

#### Executive Function (EF) Coding of Professionalism Behaviors

Professionalism behaviors were coded using a structured EF framework informed by the applied executive skills model described by Dawson and Guare [12]. While cognitive neuroscience commonly conceptualizes executive function around three core processes (e.g., inhibitory control, working memory, and cognitive flexibility) these core processes are often operationalized within educational settings into broader executive skills that are more readily observable in everyday performance, including planning/prioritizing, organization, task initiation, metacognition, emotional control, and time management. We selected this applied framework because the objective of the study was to examine observable professionalism behaviors rather than laboratory measures of executive function. Definitions of each executive function domain are provided in Table 1.

Coding reflected conceptual alignment between professionalism behaviors and executive function domains and should not be interpreted as evidence that executive function deficits were directly responsible for the observed behaviors.The structured survey question asking participants to list up to 10 professionalism concerns generated qualitative behavioral descriptions. These narrative responses were subsequently analyzed using a structured executive function coding framework in which each professionalism behavior was assigned to one or more predefined executive function domains. Predefined EF domains included metacognition, planning/prioritizing, response inhibition, emotional control, time management, goal-directed persistence, flexibility, task initiation, sustained attention, organization, and working memory. Two coders independently reviewed each text response and assigned it to one or more EF domains based on its conceptual alignment with predefined domain definitions. Coders then met to establish consensus regarding coding assignments. When consensus could not be reached, a third coder reviewed the response and participated in the final determination. Collectively, the coders brought expertise in psychology, teaching, and learning, and executive function concepts, including their application in educational settings. Multiple EF domains could be assigned to a single professionalism concern when appropriate. Because professionalism behaviors often reflect multiple interacting cognitive processes, coders were permitted to assign more than one executive function domain to a single professionalism concern when conceptually appropriate. This approach allowed the coding framework to capture the multidimensional nature of professionalism behaviors rather than forcing each concern into a single EF category.

Descriptive analyses were conducted to calculate the total number of professionalism concerns, frequencies of EF domains, regional distributions, respondent and institutional counts, and the most common EF pairings and triplets. Pairwise and triplet co-occurrence analyses were performed to identify commonly occurring combinations of EF domains associated with professionalism concerns. Because this study was exploratory, analyses were descriptive rather than inferential. The observed executive function patterns are intended to identify potential relationships and generate hypotheses for future investigation rather than confirm associations between executive function and professionalism concerns.

#### Optional Open Ended Qualitative Analysis

Qualitative analysis was conducted on the open-ended responses provided by participants regarding additional observations and insights related to professional and unprofessional behaviors in medical education. A reflexive thematic analysis approach, as described by Braun and Clarke [13], was used to identify recurring patterns and themes within the narrative data.

Responses were initially reviewed independently by two members of the research team to achieve familiarity with the data. Initial open coding was conducted by assigning descriptive labels to meaningful segments of text without imposing predefined categories, allowing patterns and concepts to emerge directly from participant narratives. Codes were subsequently organized into higher-order themes through iterative discussion among the research team. Following completion of the thematic analysis, the qualitative themes were interpreted alongside the executive function findings to provide contextual understanding of the results.

The analysis aimed to explore how participants conceptualized professionalism concerns and to identify recurring contextual or developmental factors associated with observed behaviors. Representative themes were used to supplement and contextualize the EF coding analyses. Because the qualitative component was embedded within a mixed-methods survey, data collection was not guided by thematic saturation. Rather, all available open-ended responses were analyzed to provide contextual understanding of the quantitative findings.

## Results

### Response/Completion

Of 545 individuals who accessed the survey, 156 began it and 63 completed all items, for a completion rate of 40.4% among those who began (11.6% of all who accessed). Because recruitment used open distribution, the number of invited-eligible individuals could not be determined; a denominator-based response rate is therefore not calculable, and we report the completion funnel instead.

### Participants

Respondent demographics can be seen in Table 2. Eighty respondents provided at least one professionalism behavior and were included in the executive function coding analyses; these respondents represented 64 unique institutions. Of these, 63 completed all survey items, including the demographic questions, and constitute the denominator for the sample characteristics reported in Table 2. Regional representation was predominantly from the Southern region, which included 20 institutions. The Northeastern, Central, and Western regions each contributed responses from 6 institutions, while 1 institution was represented from the Caribbean region. An additional 25 institutions were categorized as Missing/Other because regional information was unavailable or could not be classified within the predefined categories. These responses were retained in the analyses and categorized as “Missing/Other” to maximize use of the available data. See Table 3.

**Table 2:** Characteristics of survey respondents

Completed responses (analytic sample), *N* = 63. Percentages are within the sample unless noted. Values are n (%)			
**Characteristic**	**n**	**%**	**Notes**
Primary role in institution			
Faculty	40	63.5%	
Non-faculty / administrator	23	36.5%	
Faculty level			Asked of faculty only (*n* = 40); codes pending labels
Assistant Professor	9	22.5%	
Associate Professor	21	52.5%	
Full Professor	10	25.0%	
Academic / administrative level			*Codes pending labels*
Course/Clerkship Director	10	15.9%	
Program Director	11	17.5%	
Associate Dean	18	28.6%	
Assistant Dean	13	20.6%	
Dean	1	1.6%	
Department Chair	2	3.2%	
Program Manager	8	12.7%	
Primary level of medical education			*Codes pending labels (likely UME / GME / Both)*
Undergraduate Medical Education (UME)	47	74.6%	
Graduate Medical Education (GME)	1	1.6%	
Both	15	23.8%	
Worked directly with medical trainees since 2023			
Yes	60	95.2%	
No	3	4.8%	
Sex assigned at birth			*Codes pending labels*
Male	9	14.3%	
Female	46	73.0%	
Prefer Not To Answer	8	12.7%	
Race / ethnicity			*Select all that apply; % of respondents*,* may exceed 100%*
American Indian or Alaska Native	0	0.0%	
Asian	4	6.3%	
Black or African American	2	3.2%	
Hispanic or Latino/a/x	5	7.9%	
Native Hawaiian or other Pacific Islander	0	0.0%	
White	45	71.4%	
Middle Eastern or North African (MENA)	1	1.6%	
Another race or ethnicity	1	1.6%	
Prefer not to answer	8	12.7%	
Response & Missing-Data Accounting			
Disposition	**n**	Note	
Total responses received	156		
Completed (analytic sample)	63	*Full demographic data*	
Started but not completed	93	*Dropped out before demographic items; excluded from* Table 1	

Missing-data treatment: Missingness was overwhelmingly unit non-response (93 respondents who started but did not complete the survey). These have no demographic data and are excluded from the respondent-characteristics table; the analytic sample is the 63 completed responses

Within completers there was no item-level missing data on the variables shown. Blank “faculty level” cells reflect survey skip logic (question shown to faculty only) and are treated as not applicable, not missing

Rows labeled “code … (label to confirm)” are stored as numeric codes in the export with no codebook in the file. Provide the answer-option labels and these will be replaced with the true category names

Source: QuestionPro export “QuestionProShorten.xlsx”, Raw Data sheet; report timestamp 2025-10-09

**Table 3:** Most frequently identified executive function domains by Region

Region	Top EF Domain	*n* (%)	Second Most Common EF Domain	*n* (%)
Southern	Planning/Prioritizing	37 (17.9)	Metacognition	35 (16.9)
Western	Response Inhibition	15 (17.9)	Emotional Control	15 (17.9)
Northeastern	Planning/Prioritizing	12 (19.7)	Metacognition	11 (18.0)
Central	Planning/Prioritizing	10 (20.4)	Metacognition	9 (18.4)
Caribbean	Metacognition	4 (26.7)	Planning/Prioritizing	3 (20.0)
Missing/ Other	Metacognition	44 (19.0)	Planning/Prioritizing	34 (14.7)

### Coding

Using the EF framework, two investigators independently coded all 425 professionalism concerns identified and agreed on 229 concerns prior to consensus discussions, resulting in an initial agreement percentage of 53.9%. Discrepancies were subsequently resolved through discussion, with a third reviewer adjudicating unresolved cases.

### Executive Function (EF)

Across responses to the structured professionalism behavior question, participants reported 425 professionalism concerns, with a mean of 6.39 concerns per respondent (SD = 2.69, median = 6, range 1–10). Because multiple executive function domains could be assigned to a single professionalism concern, the 425 professionalism concerns generated 646 EF coding occurrences, corresponding to an average of 1.52 EF domains per professionalism concern. See Table 4 for examples of professionalism behaviors mapped to executive function domains. Metacognition was the most frequently identified EF domain (*n* = 116, 18.0%), followed closely by planning/prioritizing (*n* = 110, 17.0%), response inhibition (*n* = 93, 14.4%), and emotional control (*n* = 87, 13.5%). See Table 5. Additional frequently identified domains included time management (*n* = 61, 9.4%), goal-directed persistence (*n* = 54, 8.4%), and flexibility (*n* = 47, 7.3%). Less frequently identified domains included task initiation (*n* = 35, 5.4%), sustained attention (*n* = 21, 3.3%), organization (*n* = 21, 3.3%), and working memory (*n* = 1, 0.2%). Collectively, metacognition, planning/prioritizing, and response inhibition accounted for more than half of all EF occurrences identified in the dataset (Table 5).

**Table 4:** Examples of professionalism behaviors mapped to executive function domains

Professionalism Behavior	Associated Executive Function(s)
Repeatedly leaving clinical shifts without following up on patient issues or communicating with supervising faculty because “it’s 5 pm”	Goal-Directed Persistence; Metacognition; Planning/Prioritizing; Cognitive Flexibility
Looking at a phone or computer screen during class, conferences, or professional interactions instead of attending to the activity	Sustained Attention; Response Inhibition;
Arriving late to class, clinic, or other required activities	Planning/Prioritizing; Time Management; Goal-Directed Persistence; Metacognition
Avoidance	Task Initiation
Failure to complete credentialing requirements by established deadlines	Planning/Prioritizing; Time Management; Organization
Student does not follow instructions	Working Memory
Disrespectful communication (e.g., speaking in a dismissive, rude, or sarcastic manner to patients, peers, staff, or faculty)	Response Inhibition; Emotional Control;
Displaying entitlement by expecting preferential treatment or resources (e.g., parking, scheduling, assignments) and reacting negatively when expectations are not met	Metacognition; Cognitive Flexibility
Disparaging or disrespectful teaching evaluations used for venting or personal attacks rather than constructive feedback	Response Inhibition; Emotional Control;
Inappropriate clothing during patient care activities (e.g., overly casual, unclean, or inconsistent with institutional expectations)	Metacognition
Invoking “wellness” to avoid required educational activities that are not preferred	Metacognition; Goal-Directed Persistence
Expectation that curriculum should align exclusively with third-party resources rather than institutional educational objectives	Cognitive Flexibility

**Table 5:** Frequency of executive function domains by geographic region and institutional representation

Region	Metacognition	Planning/ Prioritizing	Response Inhibition	Emotional Control	Time Management	GDP	Flexibility	Task Initiation	Sustained Attention	Organization	Working Memory
Southern 20 (31.3)	35 (17.0)	37 (18.0)	26 (12.6)	29 (14.1)	24 (11.7)	17 (8.3)	14 (6.8)	10 (4.9)	6 (2.9)	7 (3.4)	1 (0.5)
Western 6 (9.4)	13 (15.5)	14 (16.7)	15 (17.9)	15 (17.9)	7 (8.3)	7 (8.3)	4 (4.8)	3 (3.6)	2 (2.4)	4 (4.8)	0 (0.0)
Northeastern 6 (9.4)	11 (18.0)	12 (19.7)	10 (16.4)	9 (14.8)	6 (9.8)	5 (8.2)	3 (4.9)	2 (3.3)	1 (1.6)	2 (3.3)	0 (0.0)
Central 6 (9.4)	9 (18.4)	10 (20.4)	8 (16.3)	7 (14.3)	5 (10.2)	4 (8.2)	2 (4.1)	2 (4.1)	1 (2.0)	1 (2.0)	0 (0.0)
Caribbean 1 (1.6)	4 (26.7)	3 (20.0)	3 (20.0)	3 (20.0)	1 (6.7)	1 (6.7)	0 (0.0)	0 (0.0)	0 (0.0)	0 (0.0)	0 (0.0)
Missing/Other 25 (39.1)	44 (19.0)	34 (14.7)	31 (13.4)	24 (10.3)	18 (7.8)	20 (8.6)	24 (10.3)	18 (7.8)	11 (4.7)	7 (3.0)	0 (0.0)
Total 64 (100)	116 (18.0)	110 (17.0)	93 (14.4)	87 (13.5)	61 (9.4)	54 (8.4)	47 (7.3)	35 (5.4)	21 (3.3)	21 (3.3)	1 (0.2)

*Note*. *N* (%). Institutional percentages are based on the 64 unique institutions represented in the sample. Percentages within each executive function domain row are calculated relative to the total number of executive function occurrences within that region. The Total row percentages are calculated using the total number of executive function occurrences identified across the dataset (*N* = 646). Multiple executive function (EF) domains could be assigned to a single professionalism concern; therefore, frequencies represent coding occurrences rather than unique professionalism events

To better characterize these multidimensional relationships, pairwise and triplet co-occurrence analyses were conducted to identify the executive function domains that most commonly occurred together within individual professionalism concerns. Analysis of EF co-occurrence patterns demonstrated that professionalism concerns frequently involved multiple interacting EF domains. The most common EF pairings were emotional control with response inhibition (*n* = 45), metacognition with planning/prioritizing (*n* = 45), emotional control with metacognition (*n* = 42), planning/prioritizing with time management (*n* = 42), and planning/prioritizing with response inhibition (*n* = 41).

Similarly, triplet analyses identified several recurring combinations of EF domains. The most frequently identified triplet was emotional control, flexibility, and goal-directed persistence (*n* = 14), followed by emotional control, goal-directed persistence, and metacognition (*n* = 10), emotional control, flexibility, and metacognition (*n* = 9), and emotional control, metacognition, and planning/prioritizing (*n* = 7). These findings suggest that professionalism concerns often reflect interconnected challenges involving behavioral regulation, adaptability, planning, and self-monitoring rather than isolated deficits within a single EF domain.

Regional analyses demonstrated broadly similar executive function (EF) patterns across geographic areas. Institutions in the Southern region demonstrated the highest overall frequency of EF-coded professionalism concerns, particularly in metacognition, planning/prioritizing, response inhibition, and emotional control (Table 5 ). Western and Northeastern institutions demonstrated moderate frequencies with similar distributions across domains, while Central and Caribbean institutions demonstrated lower overall frequencies but maintained comparable patterns in dominant EF categories. Working memory and sustained attention were infrequently identified across all regions. Despite differences in the absolute number of EF occurrences across regions, the relative distribution of EF domains was remarkably consistent. As shown in Table 5 metacognition and planning/prioritizing were among the most frequently identified domains across nearly all regions. Although the “Missing/Other” category represented the largest proportion of institutions, its executive function profile was generally consistent with the overall study findings, with metacognition and planning/prioritizing remaining among the most frequently identified domains (Table 5).

To assess whether unclassified institutions distorted the overall findings, a sensitivity analysis was conducted using only the 415 EF codings (64.2%) from institutions assigned to a defined AAMC region. The four most frequently coded domains in this restricted sample were identical to those in the full sample (metacognition, planning/prioritizing, response inhibition, and emotional control), indicating that the overall distribution of EF domains was robust to regional classification. No inferential testing of regional differences was performed.

### Qualitative Analysis of Optional Open-Ended Responses

Separate from the structured professionalism behavior question, 30 participants provided responses to the optional open-ended survey question inviting additional observations regarding professionalism in medical education. These narrative responses underwent thematic analysis, and five major themes were identified: (1) Professionalism as a Socio-Cultural Construct, (2) Longstanding Professionalism Challenges Within Medical Education, (3) Professionalism Behaviors Consistent with Executive Function Processes, (4) Modern Professionalism Challenges, and (5) The COVID-19 Pandemic as an Inflection Point.

#### Professionalism as a Socio-Cultural Construct

Participants frequently described professionalism as context-dependent and socially constructed rather than a fixed set of universal behaviors. Respondents emphasized that behaviors must be interpreted within their broader situational context and cautioned against rigid or overly simplistic definitions of professionalism. Several participants noted that professional conduct can be expressed in different ways depending on individual communication styles, learning preferences, and evolving societal norms.

One participant reflected that, *“We should also recognize that professionalism is not a single style. The quiet*,* reflective trainee can still be fully engaged and accountable. The student who prefers electronic communication and interactions can still be highly professional if expectations are clear.”* Similarly, another respondent emphasized the importance of context when evaluating behavior, noting that *“All of the behaviors don’t happen in a vacuum. For example*,* someone may be on their phone while engaged in a professional conversation because a patient just reached out with a potentially emergent inquiry.”*

These responses suggest that educators recognize professionalism as a dynamic construct that requires contextual interpretation and flexibility in assessment.

#### Longstanding Professionalism Challenges Within Medical Education

A second theme reflected persistent challenges within the professionalism literature regarding subjectivity, hidden curricula, and inconsistent assessment practices. Participants described professionalism as highly variable, difficult to operationalize, and subject to differing interpretations among learners and faculty.

One participant succinctly summarized this challenge by describing professionalism as *“Highly variable and difficult to teach!”* Another noted that *“what one used to think would be the norm or understood as professional is not. It must be explicitly taught*,* reinforced*,* and assessed.”* Several respondents also observed that learners often possess different frameworks for understanding professional behavior, reflecting changing cultural and generational expectations.

Concerns regarding equity and bias in professionalism assessment also emerged within this theme. One respondent highlighted that learners with neurodevelopmental differences may be disproportionately labeled as unprofessional, stating that *“people with neurodiversity such as ADHD and autism are labeled as unprofessional many times when they are not and instead are being judged by neurotypical standards.”*

Collectively, it is suggested that assumptions about what constitutes professional behavior can no longer be taken for granted and that professionalism expectations increasingly require explicit instruction.

#### Professionalism Behaviors Consistent with Executive Function Processes

A third theme reflected participants’ descriptions of professionalism concerns that conceptually aligned with executive function processes. Although participants did not describe executive function directly, many of the observed behaviors were readily interpreted through the executive function framework used in this study. Respondents frequently reported difficulties related to response inhibition, emotional control, metacognitive awareness, sustained attention, goal-directed persistence, and communication. Rather than describing isolated incidents, participants often portrayed patterns of behavior characterized by poor engagement, resistance to feedback, limited self-awareness, and challenges with accountability.

Several respondents highlighted concerns regarding learner engagement and sustained attention. One participant noted that some learners *“are not engaged. Some have been observed watching videos or ordering online by attendings*,*”* while another described *“the constant lack of initiative in learning*,*”* observing that students often appeared unwilling to fully engage in educational activities and became frustrated when performance expectations were not met. These behaviors were conceptually aligned with several executive function domains. Technology distractions and difficulty maintaining focus are consistent with challenges in sustained attention, whereas reluctance to begin or persist with learning activities aligns with task initiation and goal-directed persistence. The inclusion of metacognition reflects participants’ descriptions of learners demonstrating limited awareness of how their engagement (or lack thereof) affected their own learning, performance, and professional responsibilities. Thus, rather than representing isolated deficits, these behaviors may be interpreted as involving multiple interacting executive function processes.

Difficulties with self-awareness and communication also emerged as recurring concerns. As one participant explained, *“In instances of unprofessionalism I have encountered*,* communication skills appear to play a significant role.”* The same respondent emphasized that *“self-awareness seems to me to be an important part as well.”* These observations are consistent with the prominence of metacognition in the EF coding findings and suggest that the ability to monitor one’s own behavior and understand its impact on others may be central to the enactment of professional behavior.

Participants additionally described challenges related to emotional regulation and responses to accountability. One respondent noted that *“students openly argue with faculty when attempting to hold them accountable*,*”* while another observed that learners may become hostile when expectations are not met and subsequently blame faculty for their difficulties. Such behaviors may be conceptually interpreted through EF domains related to response inhibition, emotional control, and cognitive flexibility, particularly when individuals encounter feedback, frustration, or competing demands.

Many respondents also described concerns regarding learner maturity, independence, and professional judgment. One participant reflected on a *“sense of delayed maturity”* among learners compared with previous generations, citing differences in emotional maturity, independence, and life experience. Another noted that trainees sometimes appear more focused on their own needs than those of patients, stating that *“trainees focus more on themselves and their wellness than that of the patients they are training to serve.”* These comments suggest perceived challenges in metacognitive awareness, emotional control, and the ability to balance personal needs with professional responsibilities.

Notably, entitlement emerged as the most frequently discussed behavioral concern within this theme. Participants repeatedly described learners as demonstrating unrealistic expectations, resistance to challenge, and an excessive focus on personal preferences. One respondent remarked that *“today’s students have a sense of entitlement*,* want the easy way to do everything*,* complain if there are more than one exam in a week*,* lack social skills and crumble under pressure.”* Another stated that *“the sense of entitlement is off the charts*,*”* while a third described learners entering training environments asking, *“What is in this experience for ME?”* Although entitlement is often interpreted as a personality characteristic, an EF perspective suggests that some of these behaviors may also reflect underlying challenges with metacognitive awareness, emotional regulation, and cognitive flexibility.

Taken together, these findings suggest that many commonly cited professionalism concerns may represent observable manifestations of underlying EF processes. While professionalism cannot be reduced to EF alone, participants frequently described behaviors that align with the EF domains identified, particularly metacognition, planning/prioritizing, response inhibition, emotional control, and goal-directed persistence. These observations provide additional support for considering EF as one lens through which professionalism lapses may be understood and addressed.

#### Modern Professionalism Challenges

Participants also described emerging professionalism concerns associated with contemporary educational and technological environments. Social media, digital communication, remote learning, and evolving workplace expectations were frequently cited as creating new challenges for learners and educators.

One participant commented that *“Social media has decreased everyone’s attention span*,*”* noting that learners may engage in texting or other digital activities during face-to-face interactions with faculty and preceptors. Others discussed how asynchronous communication and virtual learning environments have altered expectations regarding accessibility, flexibility, and professional boundaries.

As one respondent explained, *“Learners have become accustomed to asynchronous communication*,* flexible schedules and working from home which can have a positive impact on autonomy*,* but sometimes blurs the professional boundaries.”* Participants further noted that reduced opportunities for in-person interactions may limit observation, role modeling, and informal social learning processes that have traditionally contributed to professional identity formation.

#### The COVID-19 Pandemic as an Inflection Point

The final theme centered on perceptions that the COVID-19 pandemic represented a significant turning point in learner professionalism, socialization, and professional identity development. Participants frequently attributed changes in learner behaviors, expectations, and interpersonal interactions to disruptions experienced during the pandemic.

Several respondents described concerns that prolonged periods of remote learning reduced opportunities for socialization and professional role modeling. One participant reflected that *“These students had to learn to operate independently*,* without peer contact*,* during COVID*,* and it may have impacted their comfort level during in-person activities.”* Others suggested that developmental opportunities may have been altered during a critical period of maturation, with one respondent stating, *“I believe the pandemic altered development during an impressionable youth period and that will not be regained.”*

Participants also described shifts in expectations surrounding flexibility, accommodations, and institutional responsiveness. One respondent suggested that increased requests for exceptions and rule modifications may reflect experiences during the pandemic, noting that *“it’s a product of the Covid years*,* when everyone was just doing their best to get through and making allowances and adjustments because that’s just what needed to happen.”*

Taken together, these findings suggest that educators perceive the COVID-19 pandemic as a major contextual influence shaping contemporary professionalism challenges. Participants described effects on socialization, communication, expectations, and learner development that continue to influence educational environments beyond the immediate pandemic period.

## Discussion

This multi-institutional exploratory study suggests that professionalism concerns reported by medical educators may be meaningfully interpreted through an EF lens. Across institutions, professionalism behaviors clustered consistently around metacognition, planning/prioritizing, response inhibition, and emotional control, suggesting that these domains may represent common cognitive processes underlying many observable professionalism concerns. Importantly, these findings should be interpreted as hypothesis-generating rather than confirmatory. Rather than proposing that EF deficits cause professionalism lapses, this work suggests that EF offers a complementary framework for understanding and operationalizing professionalism behaviors within competency-based medical education.

Institutions classified as “Missing/Other” represented 25 of 64 institutions (39.1%) and 231 of 646 EF codings (35.8%), meaning the five defined regions account for 415 codings (64.2%). This proportion warrants caution in interpreting regional comparisons, and we therefore present regional distributions descriptively rather than as evidence of geographic variation. Notably, the EF domains identified within the Missing/Other group closely paralleled those observed across the defined regions: metacognition, planning/prioritizing, response inhibition, and emotional control were the four most frequently coded domains in every region as well as within the Missing/Other group, with only minor differences in rank order. This consistency suggests that the overall distribution of EF domains the study’s primary finding, computed across all 646 codings is not driven by unclassified institutions.

### Executive Function as an Operational Framework for Professionalism

One of the greatest challenges in professionalism education is translating broad competency statements into observable behaviors that can be consistently assessed and remediated. Existing professionalism frameworks describe *what* constitutes professional behavior but often provide limited guidance regarding *why* learners struggle or how educators should coach improvement. The present findings suggest that executive function may help bridge this gap by providing a language that links observable professionalism behaviors with underlying cognitive and self-regulatory processes. Rather than documenting repeated lateness simply as a professionalism concern, educators might consider whether the learner is experiencing challenges with planning, time management, task initiation, or metacognitive awareness. Instead of providing nonspecific feedback such as “be more professional,” coaching conversations can focus on concrete strategies such as planning travel time, anticipating and prioritizing competing demands, establishing routines, or monitoring punctuality. The professionalism expectation remains unchanged; however, the feedback becomes more specific, actionable, and developmentally oriented [14].

Similarly, professionalism concerns involving communication can be operationalized through specific executive function domains. A learner who interrupts patients or colleagues, responds defensively to feedback, argues with faculty when held accountable, or sends emotionally charged emails may initially appear to demonstrate poor professionalism. Through an EF lens, however, these behaviors can be understood as involving response inhibition, emotional control, and metacognitive awareness.

### Implications for Competency-Based Medical Education

One of the central goals of competency-based medical education (CBME) is to translate broad competencies into observable behaviors that can be assessed, coached, and developed over time [1–2]. Professionalism has remained particularly challenging in this regard because competency frameworks often describe the desired behaviors but provide limited guidance regarding the underlying processes that contribute to successful or unsuccessful performance. Consequently, faculty frequently document professionalism concerns using broad descriptors such as “unprofessional,” “poor attitude,” or “lack of professionalism,” which offer little direction for learner improvement or remediation [5].

The EF framework proposed in this study offers a complementary approach that may help operationalize professionalism without redefining it. Rather than replacing professionalism competencies, EF provides educators with a vocabulary for describing the cognitive and behavioral processes through which professionalism is enacted. Coaching may therefore focus on strategies such as pausing before responding, recognizing emotional triggers, considering alternative perspectives, and reflecting on how one’s communication affects patients, peers, and supervisors. This approach preserves accountability while providing learners with skills that can be practiced and strengthened over time.

The framework may also help educators address professionalism concerns that are often interpreted broadly as entitlement or lack of motivation. For example, repeated requests for exceptions to policies, reluctance to complete required educational activities, refusal to acknowledge knowledge gaps, or expecting curricular changes to align with third-party resources may reflect difficulties with cognitive flexibility and metacognitive awareness. Rather than characterizing these learners as simply “entitled,” educators might explore how the learner understands professional expectations, responds to competing priorities, and adapts to institutional standards. Such conversations can promote reflective practice while reinforcing professional responsibilities. Similarly, a learner who responds defensively to corrective feedback might traditionally receive comments regarding attitude or professionalism. An EF-informed approach would instead recognize opportunities to strengthen emotional regulation, response inhibition, and cognitive flexibility by helping the learner develop strategies for pausing, reflecting, and incorporating feedback before responding.

This shift does not lower professionalism standards or excuse inappropriate behaviors. Rather, it changes the educational response from identifying *what* the learner did wrong to understanding *which skills may require further development* while maintaining accountability for professional expectations. Such an approach aligns closely with the developmental philosophy of competency based medical education, which emphasizes continuous growth, formative assessment, coaching, and individualized learning plans rather than solely summative judgments of performance.

The co-occurrence findings from this study further support this perspective. Professionalism concerns rarely mapped to a single EF domain, suggesting that professional behaviors often emerge through multiple interacting cognitive processes. Accordingly, remediation efforts may be more effective when they address combinations of executive skills rather than isolated behaviors. This perspective may allow professionalism coaching to become more individualized, developmentally appropriate, and aligned with the broader goals of competency-based assessment.

### Implications for Faculty Development

The qualitative findings highlight another longstanding challenge within medical education: faculty often struggle to define professionalism consistently, distinguish contextual factors from learner behaviors, and provide feedback that is both specific and actionable [1–3]. Participants described professionalism as highly variable, difficult to teach, and increasingly influenced by changing societal expectations, technology, and learner experiences. These findings suggest that improving professionalism assessment requires not only better learner support but also enhanced faculty development.

An EF-informed framework may provide faculty with a practical heuristic for observing and discussing professionalism concerns. Rather than assigning broad labels to learners, educators can first identify the observable behavior, then consider which executive function processes may be contributing to that behavior before determining an appropriate coaching strategy. Importantly, this framework is not intended to diagnose executive function deficits or infer neurocognitive impairment. Instead, it provides a structured approach for organizing observations, asking more targeted questions, and delivering behaviorally specific feedback.

The qualitative findings also emphasize that professionalism is fundamentally context dependent. Respondents described situations in which identical behaviors could have different professional meanings depending on environmental circumstances, communication preferences, or learner characteristics. Consequently, professionalism assessment should move beyond determining whether a behavior is inherently professional or unprofessional toward evaluating observable behaviors within their clinical and educational context.

Such an approach may also improve consistency across evaluators. Anchoring assessments to observable behaviors and shared EF terminology may reduce reliance on subjective judgments while preserving professionalism as a complex socio-cultural construct. The decision not to provide respondents with a predefined professionalism framework was intentional. The variability observed in educators’ descriptions reflects the reality that professionalism remains context dependent and operationalized differently across institutions, reinforcing the need for complementary frameworks that can support more consistent interpretation of observed behaviors. Rather than replacing existing professionalism frameworks, EF offers a structured way to operationalize professionalism by making feedback more specific, coaching more targeted, and assessment more transparent. Ultimately, the goal is not to change what professionalism means, but to improve how educators recognize, discuss, and support its development across undergraduate and graduate medical education.

### Implications for Assessment

The findings also raise important questions regarding how professionalism should be assessed. Current professionalism assessments frequently rely on global judgments that may vary substantially across observers. An EF-informed approach does not replace professionalism standards but may improve assessment by anchoring observations to specific behaviors and associated cognitive processes. Such an approach could improve feedback specificity, facilitate longitudinal coaching, and potentially reduce variability in assessment across faculty.

Consequently, the EF framework may have implications for equity and inclusion. Professionalism assessments have historically demonstrated disparities across gender, race, and cultural groups [6]. EF varies across individuals, including those who are neurodivergent [15]. Anchoring assessments in observable cognitive and behavioral processes may reduce variability in interpretation and decrease reliance on culturally subjective judgments. At the same time, professionalism expectations themselves must remain flexible and inclusive, recognizing that professional behavior is socially constructed and context dependent [1, 2].

### The Context: COVID-19

A particularly salient qualitative finding was the perception that the COVID-19 pandemic represented a major inflection point in professionalism development. Participants repeatedly described changes in learner behavior, socialization, communication patterns, and expectations that they attributed to the pandemic. Respondents noted that many learners completed critical developmental periods in relative isolation, with reduced opportunities for in-person learning, professional role modeling, and peer interaction. Several participants suggested that these experiences may have affected communication skills, emotional maturity, accountability, and comfort within professional environments. While these observations are inherently subjective, they align with developmental literature demonstrating that EF development is influenced by environmental experiences and social interactions [8, 9]. The prominence of COVID-related explanations within participant narratives suggests that educators increasingly interpret professionalism concerns within broader developmental and contextual frameworks rather than solely as individual learner shortcomings.

Ultimately, professionalism and executive function answer different educational questions. Professionalism defines the standards expected of physicians, whereas executive function offers one framework for understanding how those standards are enacted in practice. Used together, they may enable educators to move beyond identifying professionalism concerns toward providing more specific, developmentally informed coaching and assessment.

Importantly, operationalizing professionalism through executive function does not redefine professionalism or reduce it to neurocognitive processes. The qualitative findings clearly demonstrated that professionalism is a socio-cultural construct shaped by context, institutional expectations, evolving societal norms, and interpersonal relationships. Many of the behaviors described by participants likely emerge through interactions between executive function and environmental influences including organizational culture, workload, burnout, psychological distress, supervisory relationships, and the hidden curriculum. For example, difficulties with emotional regulation or planning may be exacerbated by chronic fatigue or excessive workload, while institutional norms may reinforce or discourage particular professional behaviors. An EF-informed framework therefore should be viewed as a complementary interpretive model that helps educators describe *how* professional behaviors may be enacted rather than *what* professionalism means. By linking professionalism concerns to observable executive processes, educators may be able to provide more consistent assessment, more targeted coaching, and more meaningful developmental feedback while preserving professionalism as a complex competency within competency-based medical education.

## Limitations

There are several limitations that should be noted. First, this study was exploratory and descriptive. The EF coding framework has not yet been formally validated, and causal interpretations cannot be made. Second, professionalism concerns were interpreted through an EF framework developed by the investigators, introducing potential subjectivity in coding. Although structured coding procedures were used and all coders had a background in psychology and learning, alternative interpretations may exist. The dataset relied on reported professionalism concerns rather than direct learner assessment. Respondents spanned 64 institutions across the United States. The completed sample was predominantly White (71.4%) and comprised 63.5% faculty and 36.5% administrators (see Table 2). As with any voluntary, self-selected survey, educators with a particular interest in professionalism may have been more likely to respond, which together with the modest completion rate limits generalizability. The study therefore reflects educator perceptions and documentation practices, which may themselves contain institutional or cultural biases. Findings should be interpreted as exploratory rather than population-representative.

An additional limitation relates to regional representation. Although respondents represented 64 institutions, 25 institutions (39.1%) could not be categorized into an AAMC-defined geographic region and were therefore classified as “Missing/Other.” This substantial proportion of uncategorized institutions may have influenced the observed regional distributions of executive function domains and should be considered when interpreting regional comparisons. Consequently, the regional analyses should be viewed as descriptive and hypothesis-generating rather than definitive representations of geographic variation.

Several defined regions also contained very few units; the Caribbean region, for example, comprised a single institution. In addition, faculty-to-administrator composition was not balanced across regions, so apparent regional differences may reflect respondent role rather than geography. Because no inferential testing of regional differences was performed, the regional findings should be read as descriptive and hypothesis-generating. Future work should collect standardized institutional identifiers at the point of data collection to reduce the number of unclassifiable cases.

Lastly, the optional narrative responses should be interpreted within the context of the study design. Because the open-ended responses were collected as part of a broader survey rather than through iterative qualitative sampling, thematic saturation was not an objective of the study. Consequently, the thematic analysis of the optional open-ended responses is intended to provide contextual insights rather than a comprehensive description of educator perspectives.

## Future Research

Future research should determine whether executive function domains prospectively predict professionalism concerns or whether they simply provide a useful interpretive framework for understanding observed behaviors. Longitudinal studies integrating direct measures of executive function with professionalism assessments will be important for distinguishing explanatory utility from causal mechanisms. Although informed by established executive function theory, the coding framework was developed for this study and has not yet undergone formal psychometric validation. Future work should evaluate its construct validity, reliability, and applicability across diverse educational settings.

Future research is needed to explore how an EF informed approach can be implemented effectively, feasibly, and responsibly within medical education environments. Importantly, the EF framework is intended to function as a heuristic to support more structured observation, feedback, and coaching rather than as a diagnostic or neuropsychological tool. As such, future studies should examine how educators can apply the framework while maintaining focus on observable behaviors and avoiding inappropriate neurocognitive labeling of learners. Not all professionalism concerns can be meaningfully interpreted through an EF lens. Some behaviors may reflect broader issues such as mental health conditions, substance use, or structural barriers within training environments. In such cases, the EF lens may help identify when additional evaluation or referral is appropriate, but it should not be viewed as a substitute for clinical assessment or institutional processes.

Additional work is also needed to evaluate faculty development strategies that support implementation of the EF framework. Future studies may explore training approaches that help educators recognize recurring behavioral patterns, apply shared terminology consistently, and maintain attention to contextual and equity-related factors during assessment and feedback processes. Research should further examine how EF-informed language influences documentation practices, learner perceptions of feedback, and the quality of coaching conversations related to professionalism development.

Moreover, because the EF framework is intended to augment existing observations rather than create additional layers of assessment, future research should evaluate its practical integration into current professionalism evaluation systems. This may include development and validation of structured tools, observation guides, or faculty support resources designed to streamline implementation while minimizing additional time burden for educators.

Finally, longitudinal studies are needed to determine whether EF-informed approaches improve professionalism assessment consistency, learner engagement with feedback, remediation outcomes, and equity within medical education. Such work will be essential in determining the educational utility and limitations of this framework across diverse training environments.

## Conclusions

Professionalism remains an essential but persistently difficult competency to operationalize and define within medical education. This multi-institutional mixed-methods exploratory study suggests that professionalism concerns may cluster around identifiable EF domains, particularly metacognition, planning/prioritizing, response inhibition, and emotional control.

Reframing professionalism through an EF lens may provide a more observable, developmentally aligned, and equitable framework for assessment and feedback. This approach does not redefine professionalism, nor does it offer a complete or empirically validated solution. Instead, it provides a structured way to connect abstract expectations with observable behaviors, while remaining attentive to developmental, contextual, and sociocultural factors. An EF-informed approach offers one way of interpreting many professionalism behaviors through observable cognitive and behavioral processes. Although this framework may improve how educators describe and coach professionalism concerns, it should not be interpreted as demonstrating that executive function deficits directly cause professionalism lapses. The EF lens should be viewed as one possible approach among many, rather than as a definitive solution.

Although preliminary, these findings support further exploration of EF as a complementary framework for professionalism assessment, coaching, and remediation in medical education.

## Supplementary Information

Supplementary Material 1.

## Declarations

### AI-Assisted Figure Development

Google NotebookLM was used to assist in the development and visual presentation of the infographic included in this manuscript as Figure 1. The content and underlying information were provided and reviewed by the authors. The authors reviewed and edited the final infographic for accuracy and take full responsibility for its content.
