# Supplementary material for: Reframing Professionalism Through Executive Function: A Multi-Institutional Analysis of Professionalism Lapses in Medical Education
Source: Med Sci Educ. 2026 Oct 14;36(6):2851–66. doi: 10.1007/s40670-026-02912-3 (PMC13759876; doi:10.1007/s40670-026-02912-3)
Supplement: Supplementary file 1 [file 40670_2026_2912_MOESM1_ESM.docx]

Study Title: Survey on Observed Professional and Unprofessional Behaviors in UME and GME McGovern Medical School at UTHealth Houston Principal Investigator: Asia Bright, PhD Contact Number: 713-500-5123 You are being invited to be in this research study because you are a faculty members and/or medical administrator who has a direct role in the education, supervision, or support of medical students and/or residents. You are affiliated with an accredited undergraduate medical education (UME) or graduate medical education (GME) program and have had direct engagement with learners in a professional capacity since 2023. Your participation is voluntary and you can stop at any time without penalty. We are doing this exploratory research study to examine examples of professional and unprofessional behaviors that medical faculty and administrators witness among medical trainees. If you agree to participate in this study: You will be asked to fill out a questionnaire about your encounters with professional and unprofessional behaviors in undergraduate medical education and/ or graduate medical education.  It will take a maximum of 10 minutes to answer the questions. You do not have to answer any questions that you do not wish to answer. There is no risk associated with this project and no personal information will be gathered during this study. You may not receive any benefits from participating in this study. There are no costs to you and you will not be paid to take part in this study. You will not be personally identified in any reports or publications that may result from this study.  Any personal information about you that is gathered during this study will remain confidential to the extent of the law. You can refuse to answer any questions asked or written on any forms.  Your participation in this study is voluntary.  A decision not to take part in this study will not change the services available to you from the PI or study staff.  If you have any questions about this project, please contact Dr. Asia Bright at 713-500-5123. Your consent to take part in this study is completing the questionnaire. This research project has been reviewed by the Committee for the Protection of Human Subjects (CPHS) of the University of Texas Health Science Center at Houston, HSC-MS-25-0528. For any questions about your rights as a research subject, please call CPHS at (713) 500-7943.

1. I have read and understood the consent information and agree to participate in this study.
2. I do not agree to participate in this study.

What is your primary role in your medical institution?

1. Faculty
2. Medical Administrator

What level of faculty?

1. Adjunct Professor
2. Assistant Professor
3. Associate Professor
4. Full Professor

Please indicate your academic or administrative level:

1. Course/ Clerkship Director
2. Program Director
3. Associate Dean
4. Assistant Dean
5. Dean
6. Department Chair
7. Program Manager
8. Other __________

Primary level of medical education you work with:

1. Undergraduate Medical Education (UME)
2. Graduate Medical Education (GME)
3. Both

Have you worked directly with medical trainees since 2023?

1. Yes
2. No

Please list up to 10 professional behaviors you have observed among learners. For each behavior, indicate whether it was observed in UME, GME, or both. It is acceptable if you are unable to provide a list of 10. Please be as detailed as you would like. Example:  Student consistently arrives to class early - UME1

 2

 3

 4

5

 6

7

 8

9

10

Please list up to 10 unprofessional behaviors you have observed among learners. For each behavior, indicate whether it was observed in UME, GME, or both. It is acceptable if you are unable to provide a list of 10. Please be as detailed as you would like. Example: Tone of voice in email is rude- Both 1

 2

 3

 4

5

6

 7

8

 9

 10

[Optional] Please share any additional observations or insights that may be helpful in understanding professional and unprofessional behaviors in medical education.

What sex were you assigned at birth, on your original birth certificate?

1. Male
2. Female
3. Prefer not to answer

How do you describe your race/ ethnicity ? (Select all that apply)

1. American Indian or Alaska Native
2. Asian
3. Black or African American
4. Hispanic or Latino/ a/x
5. Native Hawaiian or other Pacific Islander
6. White
7. Middle Eastern or North African (MENA)
8. Another race or ethnicity
9. Prefer not to answer

To prevent duplicate submissions from the same department/ school, please indicate: Institution Name:

Department or Program Affiliation:
